# Cure of Bites and Stings

**Published:** 1878-11

**Authors:** 


### CURE OF BITES AND STINGS.

Almost all these are destructive from their acid nature: consequently the cure is an alkali. Spirits of Hartshorn is one of the strongest, and in almost every house, and you have only to pour out some into a tea-cup, and dabble it on the wound with a common rag; relief is almost instantaneous. But suppose you have no Hartshorn, well, then, Saleratus is an alkali, every trifling lazy cook in the land has it, we are daily eating ourselves into the grave by its extravagant use, and the use of half a thimble-full in a week is extravagant. Moisten it with water, and use as the Hartshorn. If you have no Saleratus or Soda, pour a teacup of boiling water on as much wood ashes, stir it, and in a few moments you will have an alkali. The ley of ashes will answer a good purpose while the physician is coming. Remember the principle, the bite is an acid, the cure is an alkali.

Have we not before now looked with wonder on the old negro, who ran out when the wasp's sting made us "holler," caught up "*three kinds*" of weed, rubbed the part well, and in five minutes we were happy in the complete relief. But why "*three*" kinds of weed? Why, in the first place, you know "three" and all its multiples are mysterious numbers; and then again, you can scarcely gather up three kinds of plants anywhere, one of which will not have more or less of alkali in it. If men were only to gather up *principles* instead of *specifications*, how much easier it would be to know a great deal, and to apply our knowledge successfully to the practical purposes of life.

